# Impact of women’s participation on village savings and loan on children’s nutritional diversity in rural Chimanimani in Zimbabwe

**DOI:** 10.4102/jamba.v13i1.1043

**Published:** 2021-10-28

**Authors:** Kudakwashe A. Mbiro, Thabo Ndlovu

**Affiliations:** 1 Institute of Development Studies, Faculty of Commerce, National University of Science and Technology, Bulawayo, Zimbabwe

**Keywords:** village savings and loan, participation, health, nutritional diversity, child care, women

## Abstract

The prevalence of malnutrition in children below the age of 5 years in rural Zimbabwe, resulting from low income and the inability to meet food and medical expenses, marks huge disparities between rich and poor households. Efforts to improve child health and nutrition status culminated in different strategies being employed, chief amongst them is the enhancement of access to financial capital for vulnerable communities through improved women participation in village savings and loan (VSL). The study sought to examine the influence of women’s participation in VSL and its impact on child health and nutrition in rural Chimanimani. Arnstein’s Ladder of Participation and the United Nations Children’s Fund’s (UNICEF) framework anchor discussions on the participation of women, VSL financial resources usage by women, contribution of VSL to food diversity for children and ultimately to food consumption patterns before and during women’s participation in VSL. The study was largely qualitative and explored the descriptive research design to collect data by using semi-structured questionnaires and in-depth interviews. The findings show that the majority of participants used funds from VSL to purchase food, invest in income-generating activities and finance medical expenses. Village savings and loan contribute to an increase in food consumption score and meal dietary diversity for children, and at the same time it improved child care, health and nutrition in the rural Shinja community. The study concluded that genuine participation of women in VSL positively influenced the improvement in children’s health and nutritional diversity and that the VSL model is a multifaceted tool, which can be intertwined with other interventions to contribute to the attainment of the sustainable development goals.

## Introduction

In Africa and anywhere in the world, access to credit is a challenge as most households fall short of the required collateral (assets pledged by the borrower as security for repayment of a loan and in case of failure to pay it is forfeited by the lender) (Booth & Lena 2006). Lack of collateral by rural households is corroborated by Yunus that financial institutions pronounce a death sentence on the poor when they turn down the poor as unworthy of credit, imposing a financial apartheid (Yunus 1999, cited in Beyene & Dinbabo 2019). Against this background, Ngegba, Kassoh and Sesay (2016) indicate that whilst many banks and microfinance institutions in Africa provide valuable services to the poor, they are most successful in urban settings, where they charge high interest rates and the costs of reaching clients is low. Critical to note is that the majority of poor people in rural areas and in urban slums receive little or no financial services at all (Ngegba et al. 2016). This corroborates well with Harris’ (2009) view that for over 30 years since the start of the microfinance revolution, poor women who live in many rural areas and urban slums in Africa face difficulties in gaining access to useful microfinance products. Village savings and lending associations (VSLAs) have become one of the major sources of funding for marginalised groups, including women (Theophilus & Paul 2019). Village Savings and Lending Associates (2018), in one of its studies confirms that the VSLA model is common in approximately 75 nations in Latin America, Asia and Africa, with women comprising 78% of the membership. Village savings and lending associations pioneered by CARE International (Theophilus & Paul 2019) are largely self-managed associations, which provide members with a secure means to keep their money and access loans and emergency support resources (Ksoll et al. 2015).

The challenges in access to credit led CARE International to initiate a village savings and loan (VSL) model in Niger in 1991 (CARE 2017b). According to Abubakari, Sadik and Keisan (2014), one of the interventions to address household food consumption patterns entailed supporting women with microfinances. Furthermore, Abubakari et al. (2014) suggest that about 70% of women’s income is spent on acquiring household food, bringing to light the need for women participation in VSL. This accentuates the view that women’s access to micro-loans goes a long way in enhancing wealth status, increasing dietary diversity and improving the nutritional status of dependants (Batinge 2018).

Women’s choices differ from men; hence, placing financial resources in the hands of women has proved to lead to better outcomes in terms of the family’s education, health and nutrition (Bold, Quisumbing & Gillespie 2013). Bold et al. (2013) further accentuated that involving women in microfinance programmes improves their decision-making powers at household level and expands ownership of assets and ultimately increases expenditures on children’s education and health. A neglect of this practice contributes to chronic dietary shortcomings in children because of financial challenges and intra-household dynamics making VSL relevant in reducing the vulnerability of women (Brunie et al. 2014). Increasing income of women at household level contributes to improved child health and nutrition status as women are the cornerstone in food preparation, handling and feeding practices of children (Oniang & Mukudi 2002). Kesanta and Andre (2015) observed that funds from VSL increase accessibility, stability, availability and utilisation of nutritious food and promote investment in income-generating activities such as chicken rearing and gardening to improve the food security situation for the entire family. In responding to the question on whether participation of women in VSL contributes to improved health and nutritional status of children, the article highlights the evolution of VSL, VSL’s influence on food consumption and VSL funds’ usage followed by the impact of VSL in promoting child care, health and nutrition. The article details the study area, methodology section and VSL usage at household level, after which nutrition and dietary diversity is discussed, followed by the conclusion.

### Evolution of village savings and loan

Village savings and loan was launched by CARE Niger in 1991 to improve the financial capacity for the vulnerable communities and to engage them in micro-saving (Abubakari et al. 2014). Karlan et al. (2011) concur that VSL’s main objective is to end the challenges of offering loans to the poor by building on a rotating savings and credit association (ROSCA) model to allow participants to pool their savings and create a source of lending their funds. The most important characteristic of VSL is that it is self-managing. This is fundamental to their mode of operation and objectives, and transactions are carried out at meetings with every group member present so as to ensure transparency and accountability (Abubakari et al. 2014). Village savings and loan enables women to save and lend money and consequently access the relevant resources to build and sustain income-generating activities to invest in the health and education of their families (Allen & Staehle 2009). According to CARE (2017a), through the VSL concept women can participate in decision-making, fulfil their rights and boost their self-esteem. To understand women’s participation in VSL and its impact on child nutritional diversity, the article explored Arnstein’s Ladder of Participation and the United Nations Children’s Fund (UNICEF) framework.

Arnstein’s (ed. 1969) Ladder of Participation differentiates real participation from mere participation through the eight rungs, namely manipulation, therapy, informing, consultation, placation, partnership, delegated power and citizen control. Community development initiatives have a tendency to resort to informing, consulting and placating, with women’s voices too often drowned out and decisions made on their behalf. From partnerships to citizen control, vulnerable groups influence decisions about their future although this is rarely achieved (Rifkin & Kangere 2002). The model allowed the researchers to interrogate women’s participation in VSL and its effectiveness, and women’s accountability to child care, health and nutrition. To complement Arnstein’s Ladder of Participation, the study consulted the UNICEF framework to examine biological and socioeconomic causes of malnutrition at micro-level. The framework recognises three levels of causality corresponding to immediate, underlying and basic determinants of child nutritional status (UNICEF 1990). The UNICEF’s framework served as an instrument to show that not only is the state of the household in general important, but also the situation of mothers therein influences the care and thus the nutritional status of dependents. Women’s access and control over income resources and their knowledge and beliefs increase the quality of care, whereas a lack of control of resources, knowledge, time and social support networks decreases it (Pridmore & Hill 2009).

Fhi 360 (2015) notes that women participating in VSL commit their proceeds towards health, nutrition, food security, education, cultural events and protection against emergencies. To qualify this view, reference is made to a study in Zambia, which indicates that VSL participants apply for a loan from their group to cover household expenditures, with part of the money spent on meeting household food consumption needs (Gash 2017). Thus, VSL aid women to be financially independent by empowering them to invest in income-generating activities to meet household expenses (Fhi 360 2015) and to effectively withstand shocks such as drought (Karlan et al. 2011). The use of VSL funds to pay school fees and purchase small livestock is of critical importance (Adams, Mohammed & Kwakye 2014). In addition, evaluations show that women participants in Masvingo district use VSL funds to buy essentials for the family including clothing and food items and provide capital for income-generating activities (ENSURE 2017). Although a vast amount of literature explains the involvement of women and the deployment of funds from VSL, there is limited documentation on these uses in rural Chimanimani.

### Impact of village savings and loan in promoting child care, health and nutrition

Beyene (2018) notes that households with women participating in VSL have greater chances of achieving a better health status than households with no women participants. Participation in VSL groups lead to earning of increased income that can support the attainment of a balanced diet, food and health (Beyene 2018). Bold et al. (2013) reviewed the important linkages between women’s empowerment dimensions and nutritional outcomes with indications that women are often primary caregivers and, therefore, can directly influence their children’s nutrition through child care practices, as well as indirectly through their own nutritional status. An increase in women’s financial empowerment indicators has been associated with an increase in maternal and child health and nutrition, and conversely women’s disempowerment has shown to be associated with poorer child health and nutrition outcomes (Bold et al. 2013).

Hongo (2013) noted that the VSL programme was likely to improve access to health services for participants’ households, which was influenced by a higher level of spending on healthcare. Pronyk, Hargreaves and Morduch (2007) stated that in most cases households with VSL clients, particularly women, appeared to have children with better health and nutrition statuses compared with non-client households. Furthermore, Gash (2017) alluded that women’s participation in VSL increased the size of the mid-upper arm (MUAC) of their children and had a positive impact on body mass index (BMI) for both girls and boys. A study by Kwilasa (2017) in Madagascar revealed that there was a change in exclusive breastfeeding from 25% at baseline to 76%, stunting decreased from 41% to 36% and underweight decreased from 34% to 26%, all within a 5-year period. Akan (2017) and Silungwe (2017) attested that VSL provided a platform to raise awareness and campaigns on child care, health and nutrition. Akan (2017) concurred that in Ghana, through trainings and awareness campaigns conducted by health staff for VSL groups, health information was passed to the benefit of all involved. This research has focussed on how women’s participation in VSL for Shinja communities in rural Chimanimani promotes child care, health and nutrition.

## Study area

Chimanimani district is one of the seven districts in Manicaland with high rugged terrain, rising up to 6000 m above sea level in the east to 1600 m above sea level in the Save and Odzi valleys in the western part. The district prides itself on having all five natural agro-ecological regions found in Zimbabwe. Communities in Chimanimani survive largely on subsistence agriculture, livestock production, tourism, and cross-border trading and vending, mostly of forestry products, bananas, maize and cattle products. The district experienced cyclone Idai whose effects were so devastating to local livelihoods that many survivors’ capacity to withstand shocks eroded (United Nations World Food Programme 2019). Shinja was amongst the wards that suffered shocks from Idai, which included substantial flooding, numerous deaths and damage to infrastructure (including damage to water distribution and water infrastructure), property, crops and livestock. Prior to the cyclone, efforts to strengthen local capacities included the roll-out of VSLs; hence, the study was carried out in rural Shinja, which is one of the wards in rural Chimanimani and is found in natural agro-ecological region 4. This was an ideal study site because it is one of the underserved communities participating in VSL programmes. Shinja has a total population of approximately 1709 (813 male and 896 female), and there are 420 households (ZIMSTAT 2012). The study site had 10 VSL groups with 204 women participants (ENSURE 2018).

## Methodology

The study was largely qualitative with semi-structured and unstructured questionnaires administered in data gathering. The pragmatic research paradigm that arises out of actions and situations (Creswell 2012) informed the discussions to examine food consumption patterns for women participating in VSL. This is a philosophical underpinning for mixed methods studies; at the same time it offers the researcher the flexibility on the choice of methods and techniques of research that best meet their needs (Creswell 2012), and hence semi-structured and unstructured questionnaires were administered to gather data. Consent was sought from the provincial, district authorities and the traditional leadership of Shinja ward to conduct the study. This was supported by a letter from the university, which emphasised the need for the study to be ethical and to adhere to confidentiality issues, seek entry protocols in the ward and villages, and to give no harm assurances. The study maintained anonymity as respondents were not required to provide personal details.

The study area and key informants were purposively targeted. Key informants were drawn from the Department of Social Development under the Ministry of Social Welfare, the Family Child Health Department under the Ministry of Health and Child Care, the Department of Women Development under the Ministry of Women Affairs, community and small and medium enterprises development, traditional leaders, cluster facilitators, village health workers and World Vision (VSL officer and nutritionist officer). Using in-depth interviews, the study elicited the views of key informants on whether they considered women’s participation in VSL as having a positive effect on child care, health and nutrition.

Village savings and loan groups in Shinja community consist of both men and women, and the study targeted only women and the groups had a total of 204 women participants (ENSURE 2018). The study targeted 30% (*n* = 62) of the total population of women to generalise results to the entire population. From the list, the study further used simple random sampling in selecting 62 respondents from the targeted VSL groups to administer semi-structured questionnaires. Of the targeted 62 respondents, only 60 were interviewed. The researchers generated a simple random sample by obtaining a list of all women in each cluster from the cluster facilitator. Each woman in the VSL group was assigned a number from 1–60. The researchers randomly drew the assigned numbers from a hat to satisfy sample requirements. This was crucial because it gave all women participants an equal chance of being selected. Semi-structured questionnaires were administered by trained research assistants in collecting data from selected VSL members, with in-depth interviews used to collect information and secondary data from a wide range of people. Structured data gathered through questionnaires were analysed with the aid of Statistical Package for the Social Sciences (SPSS). Qualitative data from questionnaires and interviews were analysed by using content and thematic analyses whereby the researcher categorised verbal and behavioural data to classify, summarise and tabulate the data. Narrative analysis was also used to reformulate stories presented by the respondents.

### Ethical considerations

A letter to proceed with data collection was obtained from the National University of Science and Technology (NUST), Faculty of Commerce, Institute of Development Studies. Informed consent was sought from all study participants. The researchers also adhered to confidentiality, entry protocols in the ward and villages, gave no harm assurance and adhered to the dress code, which was appropriate in the rural community. The concept of confidentiality has its roots in the principle of respecting research participants. The respondents remained anonymous because they were not required to provide their personal details. The study also adhered to all the ethical principles of the NUST with regard to conducting research with human subjects.

## Results and discussions

### Usage of income from village savings and loan

The section examines the deployment of VSL funds by women and provides insights on how income usage has transformed at household level with the advent of VSLs. The study observes that increasing women’s income is capable of increasing their decision-making power in the household expenditures and expanding their asset base. The use of VSL funds towards household expenditures is one way, which was used to determine the level of participation of women in the VSL programme and its effectiveness towards the greatest need as shown in Table 1.

**TABLE 1:** Usage of income from village savings and loan.

Use of money	Yes		No	
	*n*	%	*n*	%
Purchased food	56	93	4	7
Medical expenses/health	34	57	26	43
Paid off debts	19	32	41	68
Productive investment	44	73	16	27
Educational expenses	40	67	20	33
Family celebration or ceremony	8	13	52	87
Savings	29	48	31	52
End of year groceries	16	27	44	73
Utensils	28	47	32	53
Building materials	8	13	52	87

Table 1 indicates that increasing women’s access to income is an effective way of enhancing commitment towards household consumption expenditures as they target areas of greatest need. The purchase of food, investments towards income-generating activities and settlement of educational expenses were identified as common expenses as these support the day-to-day survival needs of households. Interestingly, the deployment of VSL income towards savings, procurement of utensils and settlement of debts is one of the activities characterising household expenses, although not pronounced, with family celebrations and procurement of building materials being the least popular expenses. A cluster facilitator indicated how her participation in VSL has transformed her everyday life:

‘I proudly say I can now afford to buy adequate food through *mukando* [*VSL*] finances, we are also able to purchase prescribed medicine and at times invest the balance in agriculture inputs and other household assets.’ (Participant 1, female cluster facilitator, 45 years old)

This is encouraging for rural women as it reflects a vulnerable community that is determined to be self-reliant through saving and improving asset holding, which is very critical in strengthening the resilience of communities. Children require food diversity and this forces women to spend more on purchasing food to keep their children healthy. The findings concur with the study by Beyene (2018) that participation of women in VSL improved overall food consumption patterns at household level, with children managing to have three meals per day. This validates the view that when women are treated as partners in a project as indicated in Arnstein’s Ladder of Participation, trust and self-belief are entrenched, and it is at this point that communities effectively contribute to the project. Women empowerment begins at this phase as they are expected to meaningfully contribute and influence consumption patterns at household level and enhance resilience of members, especially children, as suggested by Gash (2017) that a positive change occurs in food consumption patterns for households with women participating in VSL.

In-depth discussions revealed that the VSL fund is instrumental in making participants meet adequate household food consumption levels as suggested by a cluster facilitator that ‘sharing of VSL resources is relevant in December’ (Participant 3, female cluster facilitator, 25 years old). This insinuates that the process corresponds to the period when expenditures are normally high. It enhances a sense of belonging as those participating in VSL were able to meet household expenses and at the same time managed to save finances for future use. Furthermore, in-depth discussions revealed the existence of a social fund for health emergencies, targeting members whose children fall sick during the course of the year. The findings concur with Adams et al. (2014) and Shaaban (2019) that VSL participants use their finances to upscale economic activities and enhance household health and welfare whilst others acquire business skills to diversify income sources. The behaviour displayed by women underscores the dominant narrative that they are agents of change in most localities (Morchain & Kelsey 2016); hence, more efforts should be channelled towards economic emancipation of this group to effectively transform consumption and ultimately well-being. Furthermore, it is evident that effective women participation in microfinance programmes has the propensity to influence their role in household decision-making, although with caution that it is not uniform in all contexts or in all areas of decision-making (Theophilus & Paul 2019).

### Contribution of village savings and loan on child nutrition and diversity

This study focussed on the number of meals per day, consumption of different food groups per week and nutritional diet information to examine child nutritional dietary and diversity in the rural Shinja community.

### Meals consumed by children under 5 years before and during participation in village savings and loan

Three major meals (breakfast, lunch and supper) are recommended by health specialists to be consumed by every individual in a household per day. These three major meals were used in the study to determine the consumption patterns of children with their mothers participating in VSL. Consumption of all three meals was therefore used to determine that there is an increase in food consumption pattern.

Table 2 indicates that during women’s participation in VSL the meal patterns for children under 5 years have significantly improved. The majority of respondents pointed out that the consumption patterns of children under 5 years increased from two to three meals per day. These results indicate that before women’s participation in VSL two meals that were dominant were breakfast and supper, whilst lunch was skipped as indicated by 90% of responses.

**TABLE 2:** Meals consumed by children under 5 years before and during participation in VSL.

Meal time	Breakfast (%)		Lunch (%)		Supper (%)	
	Yes	No	Yes	No	Yes	No
Before participation in VSL	92	8	10	90	93	7
During participation in VSL	100	-	98	2	100	-

VSL, village savings and loan.

Significant increase in the food consumption patterns for children under 5 years was evidenced by an increase in the number of women who were able to give their children three meals per day. In-depth discussions revealed that VSL has managed to increase the number of meals given to children through increased income and knowledge. This resonates with the findings by Brunie et al. (2014) who observe that women’s participation in VSL increase spending on food, which has been shown to lead to an increase in the number of meals consumed per day. In addition, the findings of this study are similar to a study by Fhi 360 (2015) who found that the majority of participants in VSL groups in Mozambique reported an increase in meal uptake patterns since joining the VSL.

### Frequency of consumption of different food groups before and during participation in village savings and loan

Food consumption patterns are useful in determining the likely health and malnutrition risks in a community, reflect differences in children’s eating behaviours from different households and to assess household welfare. Therefore, in line with these facts, an examination was conducted to gain an understanding of how women’s participation in VSL has influenced nutritional dietary diversity for children under the age of 5 years. Comparison of food consumption patterns before and during women’s participation in VSL was therefore used as shown in Table 3.

**TABLE 3:** Frequency of consumption of different food groups before and during participation in VSL.

Type of food	Times per week before women’s participation in VSL (%)			Times per week during women’s participation in VSL (%)		
	0–2 times	3–5 times	6–7 times	0–2 times	3–5 times	6–7 times
Cereals	2	35	63	-	-	100
Meat	73	27	0	7	85	8
Vegetables	7	13	80	-	2	98
Eggs	87	13	0	10	72	18
Legumes, nuts and seeds	55	45	0	2	85	13
Fats and oils	48	18	34	-	18	82
Milk and milk products	73	14	13	13	40	47
White tubers and roots	58	42	0	-	87	13

VSL, village savings and loan.

The results shown in Table 3 indicate that there is a significant increase in the consumption of all food types, which are meat, eggs, legumes, nuts and seeds, white tubers and roots, cereals, vegetables and fats and oils during women’s participation in VSL compared with before women’s participation in VSL. This interestingly suggests that women’s participation in VSL positively influences high food consumption patterns. Women’s participation in VSL in the long run creates a demand for agricultural products as consumption of all food types increases and improves livelihoods linked to shifts in consumption. In-depth discussions with the key informants highlight a surge in the consumption of meat by children under 5 years following women’s participation in VSL because of improved access to income and significant investment in livestock production and poultry. The increase in the consumption of meat is consistent with the findings of CARE (2015) that in Cambodia participants used funds from VSL to buy chickens and pigs for raising at home, for sale and consumption. High consumption of meat during women’s participation in VSL managed to demystify the myth that meals of children in rural areas of Zimbabwe is characterised or dominated by grains and green vegetables.

Increase in the consumption of eggs by children under 5 years is because women participating in VSL have invested in poultry projects; also those who have not invested in poultry can now purchase eggs locally by using funds from VSL. In-depth engagements with the VSL cluster facilitators indicated that before participation in VSL, women would rely only on maize for porridge, but after being part of the VSL more investment in agriculture has been witnessed resulting in different types of cereals such as finger millet, millet and sorghum grown to sustain households meals. Income-generating projects in agriculture were identified as more pronounced during in-depth discussions and as having boosted the consumption pattern of feeding children with legumes, nuts and seeds. Furthermore, investment in nutrition gardens was high as evidenced by the planting of different types of crops such as beans, groundnuts, round nuts, soya beans, cow peas, peas and butternuts. Nutrition gardens contributed to the increase in fats and oils through locally processing cooking oil and peanut butter. One of the VSL members said:

‘[*M*]*ukando* teaches us a lot, we are growing our cooking oil and peanut butter, here are the extraction machines for processing cooking oil from sunflowers and peanut butter-processing machines.’ (Participant 1, female cluster facilitator, 45 years old)

In addition, there was a moderate increase in the consumption of milk and dairy products during women’s participation in VSL mainly because of the majority of the participants’ investment in livestock production. The genuine participation of women in VSL reflects partnership and delegated power typologies where women are in charge of the planning and implementation decisions of the project. In line with the UNICEF framework on the determinants of a child’s nutritional status, the present study found that women’s participation in VSL has enhanced care, given the control of resources resulting in an increase in children’s dietary intake.

### Nutritional diet information received

Knowledge is essential in understanding what needs to be done to attain good health and nutrition for a child. Respondents confirmed receiving information on food preparation and preservation, grain and food storage, eating habits and appropriate feeding practices, and recommended agricultural practices. This information empowers women to handle diverse meal preparation requirements and deepened knowledge on the importance of nutritional diversity as shown in Table 4.

**TABLE 4:** Nutritional diet information received from village savings and loan.

Use of money	Yes (%)	No (%)
Food preparation and preservation information	83	17
Grain and food storage information	17	83
Markets with cheap food information	25	75
Eating habits and appropriate feeding practices information	75	25
Recommended agricultural practices	77	23
Any other	47	53

Table 4 indicates that VSL is an effective platform that can bring women together and teach them how to tackle and achieve the recommended balanced diet for children under 5 years. Receiving information on nutritional diet enhanced child dietary diversity and nutrition through the learning of previously unknown concepts before participating in VSL groups. Food preparation and preservation information, eating habits and appropriate feeding practices, and recommended agricultural practices were identified as common nutritional diet information received by women participating in VSL. A village health worker (also a cluster facilitator) had this to say, ‘For VSL groups, we educate and conduct competition shows, which inform on balanced diet and preparation of foods for children’ (Participant 2, female cluster facilitator, 30 years old). Grain and food storage information and markets with cheap food are the least popular nutritional diet information received by women participating in VSL, as they indirectly influence how women can promote children’s balanced diet information. Despite information on markets with cheap food being uncommon within the VSL members, it is critical because it can help them source affordable food products and increase their disposable income.

Findings of this study are similar to the findings of Ngegba et al. (2016) who revealed that through women’s participation in VSL, 66% of participants had enough stored food, 64% preserved food in different forms and 61% indicated eating properly prepared foods. In-depth discussions depicted that in VSL groups there are educational or competition shows, which they do as a group to increase knowledge on recommended food for children. Competition shows depict a balanced diet table (proteins, vitamins, carbohydrates, fats and oils), and VSL participants display prepared foods and processed food for their children, which should contain a balanced diet. Furthermore, it was revealed that in VSL groups they receive information on how to feed children with a four-star diet, for example taking proteins, vitamins, fats and carbohydrates. The received information focussed on feeding children with iron-rich foods such as liver and main four-star balanced diet, fetching drinking water from protected sources, boiling water from unprotected sources, using big cups when fetching water from the bucket, closing buckets with drinking water tightly and using milk from goats in preparing porridge. In addition, it was revealed that storage of food in tightly covered containers was encouraged to prevent flies from contaminating it, and to serve food whilst it is still hot by using clean utensils. Nutritional diet information availed through VSL platforms inspired women on how to deal with patriarchal stances without compromising children’s access to food in rural settings. They were further reminded that they are the potential leaders and capable of spreading educational information on how to feed a proper diet to their children. These findings resonate with a study conducted in Ghana by Akan (2017) who argued that VSL is a platform where women participants managed to enhance knowledge in processing foodstuffs they initially did not know how to process and identify a market where foods are cheap.

### Undernutrition cases before and after participation in village savings and loan

**FIGURE 1:**
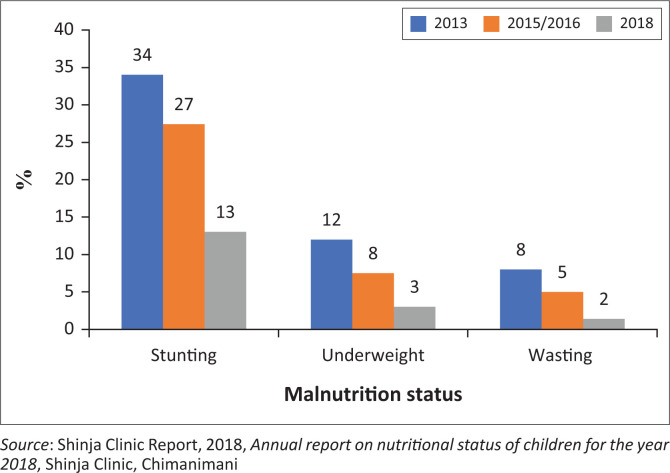
Cases of stunting and underweight in Shinja (2013–2018).

This section discusses ‘stunting’, ‘wasting’ and ‘underweight’ being the anthropometric measurements capable of revealing the levels of undernutrition in a community. The anthropometric measurements focussed on statistics for Shinja from 2013 to 2018 to examine if women’s participation in VSL has managed to reduce undernutrition levels of children as presented in Figure 1.

Figure 1 shows that from the time women have started to participate in VSL there is a decrease in the malnutrition levels in the whole ward of rural Shinja. When the anthropometric tests were conducted in Shinja in 2013, ‘stunting’ was prevalent, and ‘wasting’ and ‘underweight’ were high. During the period of 2013 and 2015–2016, ‘stunting’, ‘wasting’ and ‘underweight’ decreased slightly whilst in 2018 malnutrition cases drastically dropped with ‘stunting’ at 13%, ‘wasting’ at 2% and ‘underweight’ at 3%. The findings of this study indicate that there is a relationship between the women membership years in VSL and the improved children’s nutritional status. As women had more years in VSL, the cases of ‘underweight’, ‘stunting’ and ‘wasting’ continued to decline. Comparing anthropometric test results of children under 5 years during 2013, 2015–2016 and 2018 reveals a strong relationship of women membership years and children’s nutritional status evidenced by a wider reduction of malnutrition cases in 2018 compared with 2015–2016. The findings of this study are congruent with those of Kwilasa (2017) who indicated that women participating in VSL experienced a reduction of ‘stunting’, ‘wasting’ and ‘underweight’ in children. In addition, the findings of this study are similar to what have been observed by Abubakari et al. (2014); it was revealed that in Sisala West district in Ghana, 81% of children in households with women participating in VSLA programmes were found to be well nourished, 19% were moderately malnourished and no child was found to be severely malnourished. The study indicates that with more women participating in VSL, malnutrition cases at household and community level will drop significantly; hence, their empowerment becomes an inescapable process in contemporary settings.

## Conclusion

The study reflects that participation of women in VSL is beneficial, and that if women are given more decision-making space, they could significantly contribute to the reduction of child malnutrition cases through improved food and nutritional security. The VSL provides a platform for genuine participation for women to plan and implement decisions to diversify nutritional needs of their children under 5 years. The finding challenges government, non-governmental organisations, traditional leadership and other critical stakeholders to offer women more space in development as reflected by their ability to effectively distribute VSL proceeds across all spheres of development at a household level. The contribution by women to nutritional and dietary diversity through participation in VSL not only improves the health and well-being of children and other members of the household, but it also enhances the resilience to other health shocks. The study recommends the promotion of VSL in vulnerable areas as evidenced by their significant contribution to the reduction of stunting and underweight children in rural Shinja. Future studies should explore the usage of VSL funds and decision-making differences between men and women, as this is crticial in understanding their needs and how to complement them for the betterment of household decisions.
